# Multi-Metagenome Analysis Unravels Community Collapse After Sampling and Hints the Cultivation Strategy of CPR Bacteria in Groundwater

**DOI:** 10.3390/microorganisms13050972

**Published:** 2025-04-24

**Authors:** Kai Jiang, Lijia Ye, Chunling Cao, Gen Che, Yanxing Wang, Yu Hong

**Affiliations:** 1 College of Life Science and Technology, Inner Mongolia Normal University, Hohhot 010022, China; yelijia13158813380@163.com (L.Y.); b15248582722@163.com (G.C.); wyx168866@163.com (Y.W.); 2 Key Laboratory of Biodiversity Conservation and Sustainable Utilization in Mongolian Plateau for College and University of Inner Mongolia Autonomous Region, Hohhot 010022, China; 3 Department of Agriculture and Animal Husbandry of Inner Mongolia, Hohhot 010010, China; caochunl592186379@163.com

**Keywords:** metagenomics, Candidate Phyla Radiation (CPR), groundwater ecosystem, microbial community interactions, cultivation strategy

## Abstract

Groundwater harbors phylogenetically diverse Candidate Phyla Radiation (CPR) bacteria, representing an ideal ecosystem for studying this microbial dark matter. However, no CPR strains have been successfully isolated from groundwater, severely limiting further research. This study employed a multi-metagenome approach, integrating time-resolved sampling, antibiotic/nutrient interventions, and microbial correlation networks to unravel CPR ecological roles in groundwater and provide insights into their subsequent cultivation. Through 36 metagenomes from a groundwater system containing at least 68 CPR phyla, we revealed the time-sensitive collapse of CPR communities: total abundance plummeted from 7.9% to 0.15% within 48 h post-sampling, driven by competition with rapidly dividing non-CPR bacteria, such as members of *Pseudomonadota*. Ampicillin (100 mg/L) stabilized CPR communities by suppressing competitors, whereas low-nutrient conditions paradoxically reversed this effect. Long-term enrichment (14 months) recovered 63 CPR phyla (0.35% abundance), revealing their survival resilience despite nutrient deprivation. Correlation networks prioritized *Actinomyces*, a novel *Acidimicrobiaceae* genus, *Aestuariivirga*, *Baekduia* and *Caedimonadaceae* as potential CPR partners, providing actionable targets for co-culture trials. Here, we propose actionable recommendations spanning groundwater sampling, activation status, identification of CPR symbiotic partners, and optimization of culture conditions, which bypass traditional blind cultivation and are critical for future efforts to cultivate CPR bacterial strains from groundwater. Cultivating CPR bacteria will contribute to clarifying their diversity, ecological roles, evolutionary mechanisms, metabolic pathways, and genetic potential.

## 1. Introduction

The Candidate Phyla Radiation (CPR), first described by Brown et al. in 2015 through phylogenetic analysis of 16S rRNA gene and ribosomal proteins from metagenomes, represents a major branch of “microbial dark matter” [1,2,3]. Initially encompassing the previously recognized OD1 (now *Parcubacteria*), OP11 (now *Microgenomates*) superphyla and several candidate phyla, CPR currently includes at least 75 phyla, constituting over 15% of bacterial diversity [1,4,5,6]. Recent taxonomic revisions by the Genome Taxonomy Database (GTDB) based on genome phylogeny have reclassified CPR under the phylum *Patescibacteriota* [7,8,9,10]. These microorganisms exhibit extraordinary habitat breadth, spanning groundwater, soil, lakes, springs, extreme environments, oceans, wastewater, drinking water systems, plant rhizospheres, and animal hosts [6,11,12,13,14,15,16,17,18,19,20,21,22]. Despite their prevalence, CPR isolation and cultivation remain a formidable challenge due to their ultrasmall cell sizes, reduced genomes, limited metabolic capabilities, obligate symbiotic/parasitic lifestyles, and site-specific diversity [1,6,11,21]. To date, only a limited number of CPR strains—primarily from the *Candidatus* Saccharibacteria [23,24,25,26,27,28,29,30], *Ca*. Absconditabacteria [24], and *Ca*. Gracilibacteria [31] phyla—have been cultivated in laboratory settings, predominantly from the human oral cavity [23,24,25,26,27,30]. Notably, natural habitats like groundwater remain underrepresented, leaving key questions unresolved: what are the hosts of environmental CPR, and how do they interact? And critically, can universal cultivation strategies for these microbial dark matter lineages be developed for natural systems like groundwater?

Existing successful CPR cultivation cases rely on different methodologies, including streptomycin enrichment (selecting special antibiotic-resistant CPR strains and hosts) [23], reverse genomics (predicting cell surface proteins to cultivate selected microbiology by genomic data) [24], bait isolation (co-culturing with putative hosts) [25,26,27,30], traditional phage isolation [28], and epicPCR-directed cultivation (guided by repurposing emulsion, paired isolation, and concatenation PCR) [29]. These approaches highlight the challenges in overcoming issues such as determining host bacteria, enriching small-cell microorganisms, excluding interfering strains, and establishing specific culture media and conditions. Among these challenges, host identification is the key. Current studies demonstrate that CPR-associated host bacteria exhibit specificity, broad taxonomic diversity, and strong environmental dependency [11,32,33,34,35], which increases the difficulty of culturing CPR bacteria in the environment. Laboratory-cultivated strains provide direct evidence: *Ca*. Saccharibacteria strains have been associated with species from the class *Actinomycetes*, such as *Actinomyces*, *Schaalia*, *Cellulosimicrobium*, *Dietzia*, *Gordonia*, *Millisia*, *Nocardia*, *Rhodococcus*, *Arachnia*, and *Pseudopropionibacterium* [23,24,25,26,27,28,29,30]; Strains from the *Ca*. Absconditabacteria phylum have been found to associate with *Fusobacterium periodonticum* and *Parvimonas micra* [24], while the *Ca*. Gracilibacteria phylum has been linked to *Halorhodospira halophila* [31]. Furthermore, failed cultivation attempts for environmental CPR are also due to the uncontrolled microbial competition (e.g., fast-growing non-CPR outcompeting CPR) and inappropriate culture conditions (e.g., mismatches between in situ metabolic states and in vitro culture media).

Although research on CPR bacteria remains in its infancy, it has already revealed their intriguing potential in both human health and environmental biotechnology. Compelling evidence links CPR bacteria (*Saccharibacteria*) to oral pathologies, such as periodontitis [36], while their functional versatility is underscored by their capacity to lyse filamentous actinomycetes [28], degrade tetrabromobisphenol A in rhizosphere soil [37], and break down organochlorine pesticides under anaerobic conditions [38]. These attributes position CPR bacteria as promising candidates for wastewater treatment and soil remediation, providing innovative solutions to address anthropogenic pollution. Here, we integrate multi-metagenome datasets across different experimental conditions (e.g., post-sampling time intervals, antibiotic/nutrient perturbations) in a pristine groundwater ecosystem and coupling them with co-occurrence network analysis. This approach allows us to identify key factors driving CPR enrichment, predict potential host bacteria, and define optimal culture conditions, thereby establishing the first cultivation framework specifically designed for groundwater CPR. Our findings reveal temporal dynamics in CPR abundance post-sampling, and groundwater CPR’s abundance hinges on rapid post-sampling processing and strategic competitor suppression, while their metabolic auxotrophies and host dependencies reflect evolutionary transitions from ancestral metabolic autonomy to symbiotic lifestyles. This work provides a strong foundation for future isolation and cultivation efforts, offers valuable insights into the physiological and ecological roles of CPR bacteria in groundwater, and provides a methodological blueprint for unlocking microbial dark matter across environmental systems.

## 2. Materials and Methods

### 2.1. Sample Collection and Preparation

In May 2023 and February 2024, a series of groundwater samples were collected from a well located on the outskirts of Hohhot City, known as Hongshankou village (40°52′48″ N, 111°38′43″ E) for various purposes. Water pH, dissolved oxygen, temperature, and pH were measured using portable instrumentation. The well is a community resource, providing water for daily use by local residents. Groundwater was pumped to the sterile containers after purging the well volume and piping system. To examine the effects of post-sampling duration on the presence of CPR bacteria, twelve 25 L barrels of water were collected and stored at room temperature for varying periods (1 h, 12 h, 24 h, and 48 h, for samples GW4.1h to GW4.48h). Each storage condition had three replicates. Following storage, the samples were filtered using a 0.1 µm membrane to collect microbial cells. In a separate experiment to assess the impact of antibiotics and nutrients on CPR bacteria, 21 barrels of water, each containing 25 L, were collected. These were divided into six experimental groups and one control group, with three replicates per group. The experimental treatments included varying concentrations of ampicillin (2.5 g for samples AntibS, 0.25 g for samples AntibM, and 0.025 g for samples AntibW) and combinations of ampicillin with 25 mL of Marine Broth 2216 medium from Difco^TM^ (BD Diagnostics, Franklin Lakes, NJ, USA) (samples AntibSn, AntibMn and AntibWn). After sealing the barrels and a 48 h room temperature incubation, experimental group samples were also filtered using a 0.1 µm membrane. The control group samples (Ori) were filtered immediately after 1 h of collection without any treatment. The nutrient-enriched samples showed significant microbial growth, necessitating a reduction in filtration volume to 5 L (from the standard 25 L) to prevent membrane clogging. The samples treated with the highest concentration of penicillin (2.5 g) were stored at room temperature for an additional 14 months to examine the long-term stability of CPR (samples GW4.It). Filters were stored in a −80 °C freezer until they were later used for metagenomic sequencing.

### 2.2. DNA Extraction, Metagenomic Sequencing, and Assembly

Under sterile conditions, filter membranes were cut into small pieces and used for genomic DNA extraction via the CTAB method. The concentration, integrity, and purity of the genomic DNA were assessed using an Agilent 5400 (Agilent, Santa Clara, CA, USA). Each sample, containing 1 µg of genomic DNA, was randomly sheared into approximately 350 bp fragments using an ultrasonicator (Diagenode SA, Liège, Belgium) (15 s on, 45 s off, for a total of 6 cycles). Sequencing libraries were constructed using the NEBNext^®^ Ultra DNA library prep kit for Illumina (New England Biolabs, Ipswich, MA, USA), involving the following steps: end repair, A-tailing, adapter ligation, PCR amplification, size selection, and purification. The libraries were quantified using Qubit2.0 (Thermo Fisher Scientific, Waltham, MA, USA), and insert size distribution was assessed using an Agilent 2100 (Agilent, Santa Clara, CA, USA). Effective library concentrations, confirmed to be greater than 3nM via Q-PCR, were pooled for Illumina PE150 sequencing on Illumina NovaSeq 6000 (Illumina, San Diego, CA, USA). Metagenomic sequencing was outsourced to Beijing Novogene Bioinformatics Technology Co., Ltd. (Beijing, China), producing over 6 Gbp of raw data per sample. After sequencing, raw data were preprocessed to obtain clean data using Fastp v0.23.1 by removing reads with low-quality bases exceeding 40 bp, reads with N bases exceeding 10 bp, and reads with more than 15 bp overlaps with adapters [39]. Clean reads of each sample were assembled by using MEGAHIT software v1.2.9 with parameters setting: --presets meta-large (--end-to-end, --sensitive, -I 200, -X 400) [40,41,42,43], and Scaftigs without N were obtained by breaking from the N junction [44].

### 2.3. Bioinformatics Analyses

With default parameters, Scaftigs over 500 bp were performed to predict open reading frames (ORFs) using MetaGeneMark v2.1, and filtered out information with less than 100 nt length in the predicted results [45,46,47]. The non-redundant initial gene catalog was obtained by using CD-HIT software v4.5.8 to eliminate redundancy with the parameters: -c 0.95, -G 0, -aS 0.9, -g 1, -d 0 [48,49]. Bowtie2 was used to align the clean data to the initial gene catalog and calculate the number of reads aligned to each gene in each sample with the following parameters: --end-to-end, --sensitive, -I 200, -x 400 [42,46,50,51]. Genes with less than 3 reads were filtered out to finally obtain Unigenes for subsequent analysis. The abundance of each gene in each sample was calculated based on the number of reads aligned and the length of gene. The DIAMOND software v2.1.6 was used to blast Unigenes to the Nr database, and determined the gene number and abundance information at various taxonomic levels (phylum, class, order, family, genus, species) for each sample using the LCA algorithm [52,53,54]. The abundance of a species in a sample was determined by the cumulative abundance of genes annotated to that specific species [46,47,54]. The Unigenes were aligned to functional databases such as KEGG, eggNOG, and CAZy using the DIAMOND software v2.1.6 with the following parameters: blastp, -e 1e-5 [47,54,55,56,57,58]. Resistance Gene Identifier software v6.0.2 was used to align Unigenes to the CARD database with default parameters [59].

### 2.4. Construction of Co-Occurrence Networks

Using the Sparse Correlations for Compositional Data (SparCC) method with default settings in the Integrated Network Analysis Pipeline (iNAP) https://inap.denglab.org.cn (accessed on 13 September 2024) [60], we constructed correlation networks, respectively, based on the relative abundance matrices derived from two sets of experiments: one examining the effects of post-sampling incubation time, and another investigating the impact of adding ampicillin and low-concentration nutrients. CPR bacteria were represented at the phylum level, while other non-CPR bacteria were represented at the family level. Interaction network diagrams were visualized using Gephi v0.10.1 [61].

## 3. Results and Discussion

### 3.1. Metagenome Sequence Yield and Microbial Community

**Figure 1:**
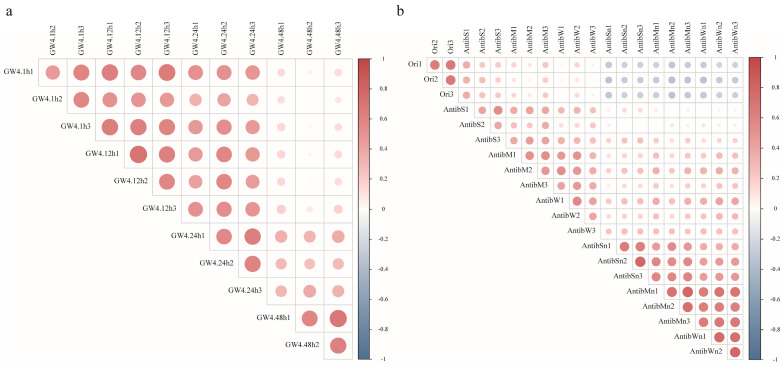
Sample correlations based on gene number. (**a**) Effects of post-sampling duration. (**b**) Impact of antibiotics and nutrients supplementation. Darker colors and larger circles indicate higher absolute correlation coefficients.

Groundwater samples were collected from a site located 30 min from our laboratory, characterized by stable physicochemical conditions (pH 6.5–7.0, salinity 0.5%, 12 °C, dissolved oxygen 8.2 mg/L). A total of 36 metagenomic samples were generated after different treatments, yielding 233 gigabase pairs (Gbp) of sequencing data (Table S1). Sequencing produced 5,423,509 scaftigs with average lengths ranging from 1032 to 1776 bp. Replicate samples exhibited strong intra-group correlations, confirming experimental reproducibility; while extended post-sampling intervals and antibiotic/nutrient perturbations significantly disrupted correlations (Figure 1). Microbial community analysis revealed dominance by *Pseudomonadota* and *Actinobacteria*, alongside diverse CPR bacteria. Notably, CPR phyla such as *Ca*. Wolfebacteria, *Ca*. Azambacteria, and *Ca*. Saccharibacteria ranked among the top 10 most relatively abundant taxa (Figures S1 and S2).

### 3.2. Temporal Dynamics of CPR Communities Post-Sampling

**Figure 2:**
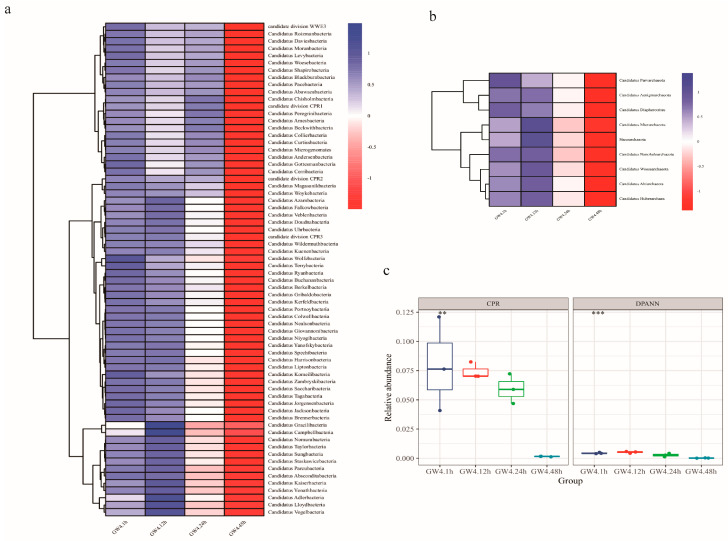
Effects of post-sampling duration on CPR and DPANN. (**a**) Relative abundance changes in CPR bacterial phyla. (**b**) Relative abundance changes in DPANN archaeal phyla. (**c**) Overall relative abundance trends in CPR bacteria and DPANN archaea. ** denotes ANOVA statistics with *p* ≤ 0.01, *** denotes ANOVA statistics with *p* ≤ 0.001.

CPR bacteria, though prevalent in environmental samples, often exhibit low relative abundances, even in groundwater [1,11,32,33,34,62]. Large volumes of groundwater must be collected and filtered to obtain sufficient biomass for analysis [1,11,32,33,34]. However, practical challenges such as the lack of on-site filtration, long distances between sampling sites and laboratories, inadequate low-temperature transport, and the considerable time consumed in water filtering process, frequently result in prolonged sample standing times. These delays may affect the detection and abundance of CPR bacteria. This highlights the critical need to analyze CPR abundance’s temporal dynamics during sampling and to optimize post-sampling processing timelines. Through metagenomic sequencing, we identified a total of 68 phyla of CPR bacteria in this groundwater. While the number of detected CPR phyla remained constant over time, both the total and individual CPR relative abundances declined sharply as standing time increased (Figure 2). One hour post-collection (GW4.1h), the total abundance of CPR bacteria was 7.9 ± 4.0%. Thirteen CPR phyla had an average abundance above 0.1%, listed in descending order: *Ca.* Azambacteria, *Ca*. Wolfebacteria, *Ca*. Saccharibacteria, *Ca*. Peregrinibacteria, *Ca*. Gottesmanbacteria, *Ca*. Chisholmbacteria, *Ca*. Harrisonbacteria, *Ca*. Magasanikbacteria, *Ca*. Parcubacteria, *Ca*. Moranbacteria, *Ca*. Woesebacteria, *Ca*. Sungbacteria, and *Ca*. Nomurabacteria. By 48 h (GW4.48h), the total relative abundance of CPR bacteria dropped to only 0.15 ± 0.025%, with *Ca*. Azambacteria (the most abundant CPR phylum) having a 36-fold decline. A parallel trend was observed in DPANN archaea, where nine phyla similarly decreased in abundance. The rapid decline of CPR abundance following sample collection underscores a critical challenge in studying these ultrasmall bacteria. We hypothesize that this decline in CPR relative abundance is due to the rapid reproduction of other microorganisms in the water, driven by increasing environmental temperatures and dissolved oxygen levels. In contrast, CPR bacteria typically possess a relatively small genome and lack stress response systems, rendering them reliant on hosts for the acquisition of essential nutrients and have an anaerobic, fermentative-based lifestyle [5,6,32,63,64]. Consequently, when environmental conditions change, CPR bacteria may not be able to adapt and reproduce as rapidly as other microorganisms. Their slower or zero replication rates under such conditions directly drive relative abundance declines. Given these findings, it is advisable to perform on-site filtration using membranes of different pore sizes or complete sample processing within 12 h to minimize the impact of environmental changes and maximize the recovery of CPR bacteria. If culturing CPR strains or their host bacteria, it is recommended to process the samples immediately if conditions permit. When immediate processing is infeasible, it is advisable to add reducing agents and store the samples at low temperatures, or employ the novel picolitre droplet technique [62].

**Figure 3:**
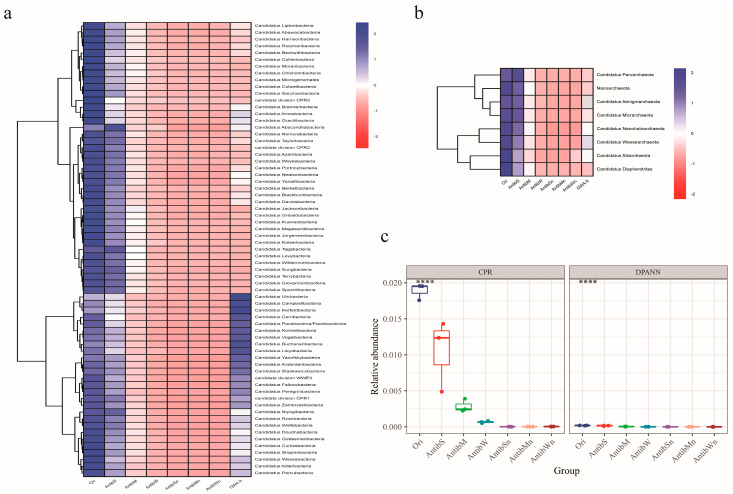
Impact of antibiotics and nutrients on CPR and DPANN. (**a**) Relative abundance changes in CPR bacterial phyla. (**b**) Relative abundance changes in DPANN archaeal phyla. (**c**) Overall relative abundance trends in CPR bacteria and DPANN archaea. **** denotes ANOVA statistics with *p* ≤ 0.0001.

Extensive evidence demonstrates that environmental microbial communities, even in stable ecosystems like groundwater, undergo compositional and functional shifts in response to temporal and environmental changes [11,14,65,66,67,68,69]. Comparative analysis of samples (GW4.1h and Ori) collected the same groundwater under identical collection and processing protocols in May 2023 and February 2024 revealed significant differences in CPR phyla abundance, diversity, and community composition (Figure 2, Figure 3, Figures S1 and S2). These fluctuations underscore the importance of sampling timing: the physiological state of CPR bacteria during collection may critically influence their cultivability.

### 3.3. Ampicillin Suppression and the Nutrient Paradox

Penicillin can inhibit growing bacterial cells, but does not affect non-growing cells [70]. Given that CPR strains grow slowly or not at all in general activation media [6,21], we designed experiments to test the effects of ampicillin and low-concentration nutrients. While large-scale enrichment of CPR cells was not observed, the addition of ampicillin alone slowed the decline in CPR relative abundance over 48 h, with higher concentrations yielding greater effects (Figure 3). At 100 mg/L, the total CPR abundance decreased only slightly, from 1.89 ± 0.09% (Ori) to 1.05 ± 0.41% (AntibS).

However, when ampicillin and low-concentration nutrients were added simultaneously, the effect was reversed. Specifically, the combination of 100 mg/L ampicillin and 1:1000-diluted 2216 medium (AntibSn) resulted in a dramatic drop in CPR abundance to 0.0016 ± 0.00019% after 48 h, a decrease of over 1000 times, with many CPR phyla approaching or below the detection limit. We speculate that while ampicillin inhibits fast-growing bacteria, ampicillin-resistant strains proliferate in the presence of nutrients, potentially secreting beta-lactamase to degrade ampicillin and further promote the growth of non-resistant strains. In this experiment, the relative abundance of the phylum *Pseudomonadota* increased from 31.87 ± 2.77% to 96.57 ± 0.34% (Ori to AntibSn). Consequently, the relative abundance of CPR strains significantly decreases.

These results indicate that simply adding nutrients and applying activation procedure favor fast-growing bacterial strains rather than CPR strains. When samples cannot be filtered on-site and must be stored for extended periods before processing, the addition of ampicillin can help maintain CPR abundance by inhibiting the growth of other bacteria. Additionally, growing evidence indicates that CPR bacteria possess a diverse array of antibiotic resistance genes [71,72,73]. These findings provide actionable targets for future cultivation strategies, as it suggests that selecting an appropriate combination of antibiotics could be a viable and promising strategy for cultivating CPR bacteria [23,74,75]. Furthermore, the ecological manifestation of CPR bacterial depletion is exemplified by their notably reduced abundance in oxygen and nutrient-replete environments (e.g., surface waters, soils) compared to groundwater systems [6,11,12,13], underscoring their competitive constraints and the broader ecosystem-level consequences of CPR decline in resource-rich niches.

To explore CPR survival and resilience, three replicate samples (GW4.It: 100 mg/L ampicillin + 1:1000-diluted 2216 medium) were incubated at room temperature for 14 months. Afterward, 63 CPR phyla were detected, and the relative abundance of CPR recovered to 0.35 ± 0.46%. Some phyla showed abundances much higher than the initial state (Ori), suggesting that CPR strains do not die during long-term culture, and may even grow slowly (Figure 3).

### 3.4. Construction of Co-Occurrence Networks and Speculation on Potential Host Bacteria of CPR

**Figure 4:**
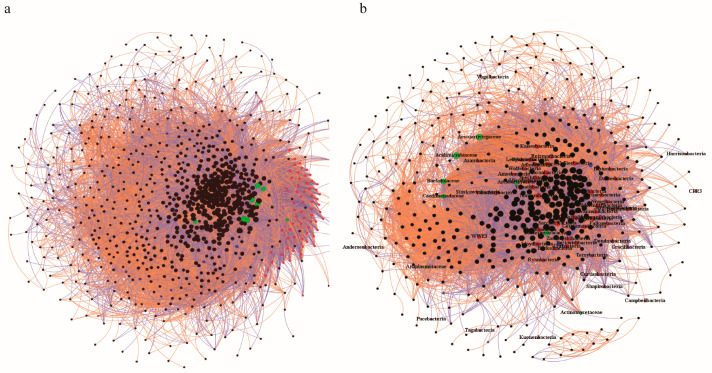
Co-occurrence networks. (**a**) Network based on post-sampling duration experiment. (**b**) Network based on antibiotic/nutrient experiment. Purple lines: negative correlations; orange lines: positive correlations. Node colors: red (CPR phyla), blue (DPANN phyla), green (family-level microbes positively correlated with CPR in both networks), black (other family-level microbes).

Using species abundance matrices from metagenomic data, two microbial co-occurrence networks were constructed, respectively. These networks depict interactions between CPR bacteria and DPANN archaea at the phylum level, while other bacteria are represented at the family level. The first network, constructed by using the data of the “post-sampling standing time” experiment, comprises 841 nodes and 42,003 edges, with 21,536 positive edges (51.27%) and 20,467 negative edges (48.73%) (Figure 4a). The second network created using the data of the “addition of ampicillin and low-concentration nutrients” experiment, includes 496 nodes and 18,966 edges, featuring 10,044 positive edges (52.96%) and 8922 negative edges (47.04%) (Figure 4b). Both networks revealed extensive CPR-associated microbial linkages. For instance, *Ca*. Daviesbacteria exhibited significant correlations, with 34 bacterial families (13 positive, 21 negative) in the first network and 69 families (26 positive, 43 negative) in the second. To minimize spurious correlations [76,77,78,79], we focused on microorganisms showing significant positive correlations with CPR across both networks. Among 68 CPR phyla detected, 42 were associated with putative partners, primarily within *Actinomycetota* (*Acidimicrobiaceae*, *Actinomycetaceae*, and *Baekduiaceae*) and *Pseudomonadota* (*Aestuariivirgaceae* and *Caedimonadaceae*) (Figure 4, Table S2). For example, *Acidimicrobiaceae* correlated with 38 CPR phyla, including *Ca*. Wolfebacteria, *Ca*. Saccharibacteria, and *Ca*. Peregrinibacteria, while *Actinomycetaceae* only showed ties to *Ca*. Saccharibacteria, *Ca*. Jorgensenbacteria, and *Ca*. Giovannonibacteria. Similarly, *Baekduiaceae* was linked to 29 CPR phyla such as *Ca*. Saccharibacteria and *Ca*. Parcubacteria, and *Aestuariivirgaceae* correlated with 38 CPR phyla, including *Ca*. Wolfebacteria and *Ca*. Saccharibacteria. The *Caedimonadaceae* family, associated with 19 CPR phyla like *Ca*. Nomurabacteria and *Ca*. Roizmanbacteria, further highlighted the diversity of potential interactions. Beyond these, specific correlations were observed between *Bacteriovoracaceae* (*Bdellovibrionota* phylum) and *Ca*. Yonathbacteria, *Arcobacteraceae* (*Campylobacterota* phylum) and *Ca*. Gottesmanbacteria, and *Anaplasmataceae* (*Pseudomonadota* phylum) and candidate division WWE3. It is noteworthy that the currently cultivated CPR strains in the laboratory belong to the *Ca*. Saccharibacteria, *Ca*. Absconditabacteria, and *Ca*. Gracilibacteria phyla [23,24,25,26,27,28,29,30,31]. While *Ca*. Absconditabacteria and *Ca*. Gracilibacteria were detected in this groundwater, no significant positive correlations were found. All host bacteria of the laboratory-cultured *Ca*. Saccharibacteria strains belong to the *Actinomycetota* phylum, specifically within four orders, six families, and ten genera, including two genera, *Actinomyces* and *Schaalia*, in the *Actinomycetaceae* family [23,24,25,26,27,28,29,30]. This *Actinomycetaceae* result aligns with our interaction network analysis (the strong positive correlation between *Ca*. Saccharibacteria and *Actinomycetaceae*), supporting the relevance of our findings. Further analysis of the *Actinomycetaceae* family in the groundwater revealed that the *Actinomyces* genus constituted 96.39% of this family, suggesting it as a potential host bacterium for *Ca*. Saccharibacteria. Further taxonomic dissection of positively correlated families revealed highly specific compositions. The *Acidimicrobiaceae* family was primarily composed of an unclassified genus (83.32%), whereas *Aestuariivirgaceae*, *Baekduiaceae*, and *Bacteriovoracaceae* each comprised a single genus (*Aestuariivirga*, *Baekduia*, and *Bacteriovorax*, respectively). The *Caedimonadaceae* family included *Caedimonas* (26.25%), *Ca*. Nucleicultrix (29.25%), and another unclassified genus (27.15%). Similarly, *Arcobacteraceae* was dominated by *Aliarcobacter* (31.64%), *Arcobacter* (28.63%), and *Poseidonibacter* (21.05%). The *Anaplasmataceae* family was predominantly *Wolbachia* (87.36%). These findings sharply narrow the scope for future host screening, prioritizing genera such as *Actinomyces*, *Aestuariivirga*, and *Bacteriovorax* for targeted isolation in this groundwater or commercial strain-based baiting strategies.

### 3.5. Metabolic Potential of CPR Bacteria in Groundwater

**Figure 5:**
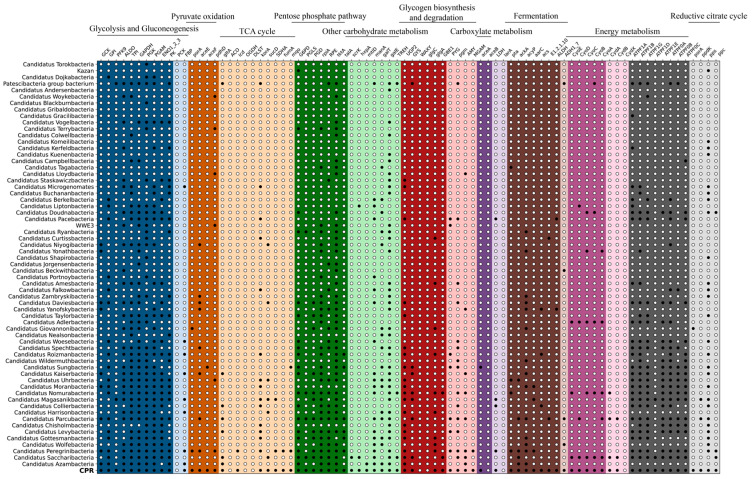
Carbon metabolic potential in groundwater. Filled symbols represent presence, while open symbols indicate absence.

Current insights into the metabolic capabilities of CPR bacteria rely heavily on metagenome-assembled genomes (MAGs) derived from metagenomic data [6,15,73,80,81,82,83,84]. While MAGs provide valuable genetic information, stringent quality thresholds (≥50% completeness, <10% contamination) [85,86,87] often exclude low-quality MAGs, limiting the scope of analysis. Given the low abundance of CPR bacteria in natural environments, obtaining high-quality MAGs remains challenging, and complete genomes are exceptionally rare. This limitation results in incomplete information being obtained from MAGs. Focusing solely on individual CPR strain genomics may overlook their collective ecological roles within a specific environment. Meanwhile, considering CPR bacteria as a group within a specific habitat and analyzing their genetic information comprehensively may offer new insights into their early states and evolutionary processes, which complements MAGs analysis. To address this, we analyzed CPR bacteria’s metabolic potential in the groundwater based on the complete metagenomic data (whole annotated information pointing to CPR in 233 Gbp of data) (detailed information in Table S3). Analyzing CPR genes as a whole, we found that they possess a complete glycolysis pathway, pyruvate oxidation pathway, pentose phosphate pathway, and glycogen biosynthesis ability. They also have a relatively complete tricarboxylic acid (TCA) cycle and gluconeogenesis pathway, lacking only the genes encoding aconitate hydratase and phosphoenolpyruvate carboxykinase, respectively (Figure 5). We did not detect the genes encoding the key enzymes of the Entner–Doudoroff pathway, phosphogluconate dehydratase and 2-dehydro-3-deoxyphosphogluconate aldolase, indicating the absence of this pathway. The glyoxylate shunt, an important replenishment pathway in microorganisms, was partially present, with the gene encoding isocitrate lyase detected, but not the gene encoding malate synthase. Regarding carbon source utilization, we identified numerous genes encoding polysaccharide hydrolases, belonging to at least 66 glycoside hydrolase families and 5 polysaccharide lyase families, covering a wide range of enzymes such as amylase, xylanase, lysozyme, licheninase, and cellulase (Table S4). Four major CO_2_ fixation pathways were analyzed; we did not find evidence of the Calvin cycle, anaerobic acetyl–CoA pathway, or hydroxypropionate pathway. However, the reverse TCA cycle pathway was relatively complete, with genes encoding key enzymes like the pyruvate ferredoxin oxidoreductase alpha subunit, pyruvate dikinase, and phosphoenolpyruvate carboxylase detected. We found key genes for the synthesis of lactate, acetate, and ethanol in CPR, indicating their ability for anaerobic fermentation. However, we did not find genes related to formic acid or methane synthesis. Accumulating evidence indicates that genomes of CPR lack the complete genetic repertoire to encode central metabolic pathways, like glycolysis, gluconeogenesis, pyruvate oxidation, and the TCA cycle [6,32,33]. Through a comprehensive comparative analysis of protein families, Méheust et al. inferred that the CPR bacterial have undergone extensive gene loss, with major genome reduction events traceable to ancient evolutionary periods [88,89]. Intriguingly, while canonical complete pathways like the TCA cycle remain undetected in extant CPR species, several genes encoding key components of these pathways are still ubiquitously present [6,32,34]. These findings lead us to speculate that in the early stages of evolution, primitive CPR cells may have had a relatively complete carbohydrate metabolism ability. Subsequently, with changes in the living environment and adopting a symbiotic or parasitic lifestyle with host bacteria, they lost genes and metabolic capabilities such as the TCA cycle and gluconeogenesis, retaining only the glycolysis and pentose phosphate metabolic pathways. As a widespread group in natural environments, CPR bacteria as a whole play an important role in promoting the global carbon cycle, particularly in the degradation of different macromolecular organic carbons and the transformation between different organic carbons. Early members of the CPR bacteria may have relied on inorganic carbon sources when organic carbon was unavailable, potentially fixing CO_2_ to sustain growth. This hypothesis is supported by studies of high-CO_2_ groundwater systems, where microbial carbon assimilation is linked to the Calvin cycle and the reductive tricarboxylic acid (rTCA) cycle [90]. Intriguingly, genes associated with CO_2_ fixation pathways have been identified in CPR MAGs [33]. Notably, the MAG *Ca*. Wirthibacter wanneri encodes a complete rTCA cycle, suggesting its potential for CO_2_ fixation [91]. Despite these genetic clues, the functional significance of these pathways in CPR bacteria remains unexplored, and no experimental or mechanistic studies have yet validated their role in carbon metabolism.

Analyzing CPR as a whole, we did not find genes related to nitrogen fixation, anammox, or nitrification. However, we detected some genes related to denitrification, including the nitrate/nitrite transporter for taking up oxidized inorganic nitrogen sources from the external environment, nitrate reductase for reducing nitrate to nitrite, and nitrite reductase for further reducing nitrite to N_2_O (Table S3). We hypothesize that CPR bacteria can use oxidized inorganic nitrogen sources such as nitrate and nitrite as electron acceptors during anaerobic respiration or sugar fermentation to produce energy under anaerobic or extremely low-oxygen conditions. Meanwhile, we found a large number of protease/peptidase, aminotransferase, and glutamate dehydrogenase genes in CPR, indicating their ability to decompose and transform organic nitrogen sources. Further analysis of amino acid synthesis ability showed that although individual cells lacked most of the essential genes for amino acid synthesis, as a whole, most of the 20 amino acids could have complete synthesis pathways pieced together, with only one or two key genes missing for threonine, methionine, cysteine, valine, and isoleucine. This result was consistent with the carbohydrate metabolism pathway, suggesting that CPR may have had the ability to synthesize amino acids in the early stages of evolution, but lost most of these genes during their long-term symbiotic/parasitic lifestyle. Analysis of sulfur metabolism-related genes revealed that there were no genes related to sulfur reduction and sulfide oxidation in the CPR group. However, we detected the genes encoding three enzymes: 3′-phosphoadenosine 5′-phosphosulfate synthase (PAPSS), thiosulfate dehydrogenase (quinone) small subunit (doxA), and sulfite dehydrogenase (cytochrome) subunit A (sorA). PAPSS can activate inorganic sulfate to adenosine sulfate and further generate 3′-phosphoadenosine-5′-phosphosulfate (PAPS), which can transfer the sulfate group it carries to other molecules, thus participating in various biosynthesis and metabolic processes [92]. DoxA can catalyze the formation of tetrathionate from thiosulfate [93], and sorA is involved in the oxidation of sulfite [94]. Overall, the CPR group in this groundwater plays an important role in promoting the cycling of nitrogen and sulfur elements, with the potential for ammonification, denitrification, sulfate assimilation, and sulfite oxidation.

In this study, we obtained 80,669 CPR KEGG annotations related to the KEGG pathway, among which 2516 were associated with the pathway of peptidoglycan synthesis (map 00550), representing 3.1% of the total. When considering CPR bacteria as a whole, we found that nearly all enzymes involved in peptidoglycan monomer synthesis and peptidoglycan assembly were present, except for monofunctional glycosyltransferase. The retention of peptidoglycan synthesis-related genes indicates the significance of peptidoglycan synthesis for CPR strains. Analysis of lipid metabolism showed that CPR bacteria lack the ability to synthesize fatty acids and triacylglycerols, as well as polar lipids such as phosphatidylcholine and phosphatidylethanolamine. In terms of nucleotide metabolism, while considering CPR bacteria as a whole, we found that complete de novo biosynthesis pathways for both purines and pyrimidines could be reconstructed. CPR bacteria do not have the capacity to synthesize vitamins, compatible solutes, or utilize urea. Collectively, only the complete synthesis pathways for riboflavin, nicotinate, and nicotinamide could be reconstructed. Notably, even when considering the CPR group as a whole, we failed to reconstruct a complete electron transport chain (ETC), lending further support to the evolutionary hypothesis that key components of the ETC were lost during ancient divergence events in CPR bacteria [88,89,95]. Intriguingly, genes encoding cytochrome or ubiquinol oxidase were detected, which potentially possessed activities similar to those of catalase and peroxidase [96], enabling the decomposition of hydrogen peroxide and thus protecting cells from damage by reactive oxygen species.

### 3.6. Insights into the Cultivation of CPR Bacteria in Groundwater

Groundwater harbors a diverse array of CPR bacteria in significant quantities, making it an ideal environment for studying these microorganisms. However, isolating CPR strains from groundwater remains elusive, and, to date, no CPR strain has been isolated from groundwater due to several obstacles. One key challenge is managing the sample collection process effectively. This involves minimizing pipeline water interference and controlling the time of sampling, transportation, and sample processing. Ideally, the entire process should be completed within 12 h. If this timeframe is not feasible, adding an appropriate amount of ampicillin can help inhibit the growth of non-CPR strains. Upon collection, samples should be processed immediately using low-pore-size filter membranes for filtration enrichment or experimentation. Traditional activation methods are not recommended, as they tend to enrich non-CPR strains, complicating subsequent cultivation efforts. Our experiments confirm that even low concentrations of nutrients can promote the growth of common microorganisms, thereby reducing the relative abundance of CPR strains.

Cultivating CPR bacteria is complex because they cannot grow independently; they require symbiotic or parasitic relationships with other microorganisms. Identifying potential symbiotic partners is therefore critical. In this study, correlation networks were constructed through two sets of experiments to narrow down the screening scope and identify microorganisms positively correlated with CPR strains. Our findings align with previous studies and provide valuable references. Specifically, we identified significant positive correlations between CPR and several bacterial families, including *Acidimicrobiaceae*, *Actinomycetaceae*, *Baekduiaceae*, *Aestuariivirgaceae*, and *Caedimonadaceae*. Further analysis narrowed the focus to the genera *Actinomyces*, an unnamed new genus in *Acidimicrobiaceae*, *Aestuariivirga*, and *Baekduia*. These potential host bacteria can be obtained through purchase or targeted isolation and cultivation, offering guidance for selecting culture media in future experiments.

Analyzing functional genes and metabolic potential in CPR bacteria provided further insights for subsequent culture media and conditions. CPR bacteria contain numerous polysaccharide hydrolase-encoding genes and possess a relatively complete glycolysis and pentose phosphate pathway, suggesting that low-concentration oligosaccharides or polysaccharides could serve as suitable carbon sources. Additionally, CPR bacteria have a variety of peptidases/proteases and amino acid transporters, but they do not have the ability to synthesize amino acids, indicating a need for nutrient-rich organic nitrogen sources. Additionally, the discovery of nitrate/nitrite transporter and nitrite reductase-encoding genes implies that many CPR strains have the ability to use nitrite as a terminal electron acceptor to produce energy under anaerobic conditions. Hence, nitrite can be appropriately added to the culture medium. CPR bacteria lack the ability to synthesize fatty acids and vitamins, necessitating their addition to the culture medium. Notably, the frequent detection of zinc transporters-encoding genes implies metal cofactor dependencies, potentially being linked to their auxotrophic requirements. Therefore, appropriate amounts of zinc can be considered for addition to the culture medium. Given that CPR bacteria lack the TCA cycle pathway and a complete ETC, and considering the low dissolved oxygen levels in groundwater, subsequent cultivation should focus on anaerobic or hypoxic environments. The presence of superoxide dismutase and oxidase, but no catalase and peroxidase-encoding genes, in CPR bacteria indicates they can tolerate some oxygen, allowing for less stringent oxygen control during cultivation. Adding an appropriate amount of reducing substances or catalase to the culture medium is advisable.

To minimize interference from non-CPR strains, the host bait isolation method is recommended. This involves enriching CPR cells through membrane filtration followed by conducting “fishing” experiments using the identified potential host bacteria under optimized culture conditions.

## 4. Conclusions

This study reveals critical challenges and strategies for cultivating CPR bacteria in groundwater ecosystems. We demonstrate that CPR abundance declines rapidly post-sampling (36-fold reduction within 48 h), likely due to competitive overgrowth of faster-replicating microorganisms under shifting environmental conditions. Ampicillin supplementation mitigated CPR loss, while concurrent nutrient addition exacerbated it, highlighting the fragility of CPR communities. Metabolic profiling revealed CPR reliance on anaerobic fermentation, partial carbon/nitrogen/sulfur cycling pathways, and dependence on host-derived nutrients, supporting their symbiotic lifestyle. Two co-occurrence networks narrowed the range of potential host and pinpointed strains in *Acidimicrobiaceae*, *Actinomycetaceae*, *Baekduiaceae*, *Aestuariivirgaceae*, and *Caedimonadaceae* families as potential CPR hosts, partly aligning with prior cultivation data. We propose protocols for further cultivation of CPR in groundwater: on-site filtration within 12 h, ampicillin use during storage, and host-targeted cultivation with optimized culture media and anaerobic conditions. These findings advance CPR research by reconciling multi-metagenome data, offering actionable strategies to overcome cultivation barriers and elucidate CPR roles in groundwater ecosystem.

## Data Availability

Raw metagenomic data were deposited in the NCBI under the BioProject accession number: PRJNA1229005 (https://submit.ncbi.nlm.nih.gov/subs/sra/SUB15106585/overview) (accessed on 24 February 2025); the BioSample accession numbers: SAMN47118940 to SAMN47118951, SAMN47118953 to SAMN47118976; the accession numbers: SRR32501921 to SRR32501946, SRR32501948 to SRR32501951.

## Supplementary Materials

The following supporting information can be downloaded at: https://www.mdpi.com/article/10.3390/microorganisms13050972/s1, Figure S1: Metagenomic data overview of post-sampling duration effects. (a) Venn diagram of shared/unique genes across sample groups. (b) Boxplot of gene number differences. (c) Top 10 phylum-level relative abundances; Figure S2: Metagenomic data overview of antibiotic/nutrient impacts. (a) Petal plot of shared/unique genes across sample groups. (b) Boxplot of gene number differences. (c) Top 10 phylum-level relative abundances; Table S1: Metagenomic sequencing data; Table S2: Potential host bacteria of CPR in the groundwater; Table S3: Metabolic analysis of the CPR based on the whole metagenomic data; Table S4: Polysaccharide hydrolases of CPR identified via CAZyme annotation of complete metagenomic data.
